# Molecular monitoring of minimal residual disease in two patients with *MLL*-rearranged acute myeloid leukemia and haploidentical transplantation after relapse

**DOI:** 10.1186/2162-3619-1-6

**Published:** 2012-04-18

**Authors:** Thomas Burmeister, Mara Molkentin, Claus Meyer, Nils Lachmann, Stefan Schwartz, Birte Friedrichs, Jörg Beyer, Igor Wolfgang Blau, Gunnar Lohm, Carola Tietze-Bürger, Rolf Marschalek, Lutz Uharek

**Affiliations:** 1 Klinik für Hämatologie, Charité CBF, Hindenburgdamm 30, 12200, Berlin, Germany; 2 Diagnostikzentrum für akute Leukämie (DCAL), Zentrum für Kinder- und Jugendmedizin, Goethe-Universität, Theodor Stern Kai 7, 60590, Frankfurt, Germany; 3 Labor für Gewebetypisierung/HLA-Labor, Charité CVK, Augustenburger Platz 1, 13353, Berlin, Germany; 4 Vivantes Klinik Am Urban, Klinik für Hämatologie, Dieffenbachstraße 1, 10967, Berlin, Germany; 5 Klinik für Radioonkologie und Strahlentherapie, Charité CBF, Hindenburgdamm 30, 12200, Berlin, Germany; 6 Stem Cell Facility, Charité CBF, Hindenburgdamm 30, 12200, Berlin, Germany; 7 Institut für Pharmazeutische Biologie, Biocenter, Goethe-Universität, Marie-Curie-Str. 9, 60439, Frankfurt, Germany

**Keywords:** Minimal residual disease, Haploidentical stem cell transplantation, *MLL*, KIR, Acute myeloid leukemia

## Abstract

This report describes the clinical courses of two acute myeloid leukemia patients. Both had *MLL* translocations, the first a t(10;11)(p11.2;q23) with *MLL-AF10* and the second a t(11;19)(q23;p13.1) with *MLL-ELL* fusion. They achieved a clinical remission under conventional chemotherapy but relapsed shortly after end of therapy. Both had a history of invasive mycoses (one had possible pulmonary mycosis, one systemic candidiasis). Because no HLA-identical donor was available, a haploidentical transplantation was performed in both cases. Using a specially designed PCR method for the assessment of minimal residual disease (MRD), based on the quantitative detection of the individual chromosomal breakpoint in the *MLL* gene, both patients achieved complete and persistent molecular remission after transplantation. The immune reconstitution after transplantation is described in terms of total CD3^+^/CD4^+^, CD3^+^/CD8^+^, CD19^+^, and CD16^+^/CD56^+^ cell numbers over time. The KIR and HLA genotypes of donors and recipients are reported and the possibility of a KIR-mediated alloreactivity is discussed. This report illustrates that haploidentical transplantation may offer a chance of cure without chronic graft-versus-host disease in situations where no suitable HLA-identical donor is available even in a high-risk setting and shows the value of MRD monitoring in the pre- and posttransplant setting.

## Background

Acute myeloid leukemia (AML) is a molecularly heterogeneous disease. Around 5–10% of affected patients show *MLL* translocations and an additional 8% have a *MLL* partial internal duplication. While *MLL* aberrations have historically and generally been associated with an adverse prognosis, this view has become more differentiated during recent years and at least for some rarer *MLL* aberrations this verdict appears to be unfounded [1]. The 5 most common *MLL* aberrations in adult AML are t(9;11)/*MLL-AF9*, t(6;11)/*MLL-AF6*, t(11;19)/*MLL-ELL*, t(11;19)/*MLL-ENL*, and t(10;11)/*MLL-AF10*, together accounting for around 80% of cases [2,3]. The general recommendation for younger and medically fit AML patients is allogeneic transplantation. The only exception are the core-binding factor leukemias with either *CBFA2-ETO* or *CBFB-MYH11*, and acute promyelocytic leukemia with *PML-RARA*, if no additional high-risk features (e.g. *FLT3*-ITD) are present. Around 15–30% of patients referred for transplantation have a suitable HLA-identical sibling donor. Large donor registries can be accessed for those patients without a HLA-identical sibling donor, but the likelihood of finding a suitable donor depends strongly on the ethnic background of the patient [4]. Currently it is possible to identify a suitable HLA-identical donor in around 70% of patients of European ancestry [5]. Different options exist for those patients without a fully (10/10) HLA antigen-matched donor. Clinical trials with donors showing only a 9/10, 8/10 or even 7/10 HLA match have shown a significantly higher transplant-related mortality, mainly due to the higher incidence of severe graft-versus-host disease (GvHD) [6-8]. The Perugia group of Martelli and coworkers first introduced the concept of transplanting “megadoses” of highly purified CD34^+^ cells of a HLA-haploidentical donor in the clinical setting to overcome the HLA barrier and prevent graft failure [9,10]. To achieve an alloreactive antileukemic effect in the absence of alloreactive T-cells, donor-derived natural killer (NK) cells were infused [11].

This report describes the clinical courses of two patients with *MLL*-rearranged AML and particular high risk features (early relapse, invasive mycoses) with particular emphasis on the MRD monitoring method which was based on the quantitative detection of the individual chromosomal breakpoints in the *MLL* gene. Both patients had no suitable HLA-identical donor and thus haploidentical transplantations were performed. The immune reconstitution after transplantation in terms of B-cells, CD4^+^ and CD8^+^ T-cells and NK cells is described.

## Case presentation

### Case 1

#### Features at presentation

**Figure 1:**
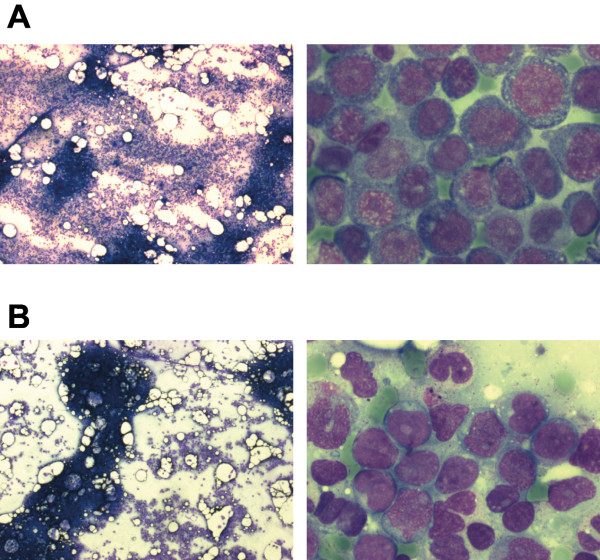
**A. Bone marrow smear at diagnosis from the first patient at two different magnifications.** The marrow shows increased cellularity by round-shaped medium-sized blasts with basophilic occasionally vacuolated sparsely granulated cytoplasm and perinuclear halos. The nuclei show 1-3 nucleoli. Peroxidase stain: 10% positive, esterase stain: 100% markedly positive, PAS stain: approx. 50% granular positive. The cytomorphology is compatible with the diagnosis of AML FAB M5a. **B.** Bone marrow smear at the time of relapse from the second patient at two different magnifications. The marrow shows an overall normal cellularity and is infiltrated by heterogenous blasts displaying oval or bean-shaped nuclei with prominent nucleoli and a moderately abundant partially granulated cytoplasm. Peroxidase stain: 20% of blasts positive, esterase stain negative. The cytomorphology is compatible with the diagnosis of AML FAB M2.

A 33-year-old male patient was diagnosed with acute myeloid leukemia. The first clinical symptoms were dyspnoea on exertion, a generalized maculopapular rash, gingival hyperplasia, and general weakness. The blood count at diagnosis revealed 52/nl leukocytes (79% blasts) a hemoglobin of 11.1 g/dl (hematocrit 0.33), and platelets of 110/nl. The LDH was 1306 U/l (normal < 248 U/l), creatinine 1.27 mg/dl, C-reactive protein 16.1 mg/dl (normal <0.5 mg/dl). Bone marrow cytology revealed an increased overall cellularity and an 80% infiltration by medium-sized blasts with basophilic cytoplasm, partial cytoplasmic vacuolisation, prominent nucleoli, and positive esterase staining, compatible with acute myeloid leukemia of the FAB M5a subtype (Figure 1). Flow cytometric analysis of the bone marrow revealed an expression of myeloid antigens and HLA-DR with aberrant CD4 and CD56 expression. Cytogenetic analysis revealed a 46,XY,t(10;11)(p11.2;q23) karyotype and molecular analysis by RT-PCR an *MLL-AF10* fusion transcript.

#### Initial treatment

The patient was randomized into arm C of the German AML2003 study after informed consent. The study protocol comprises two cycles DA (100 mg/m^2^ = 200 mg cytarabine continuously, d1-7 + 60 mg/m^2^ = 120 mg daunorubicine, d3-5) followed by 3 cycles post-remission therapy (MAC-MAMAC-MAC) with MAC = 2 × 1 g/m^2^ cytarabine d1-6 + 10 mg/m^2^ mitoxantrone d4-6 and MAMAC = 2 × 1 g/m^2^ cytarabine d1-5 + 100 mg/m^2^ amsacrine d1-5. An allogeneic transplant is recommended after completion of the first two cycles, if a donor is available. However, no HLA-identical sibling or unrelated donor could be identified in this case.

The inital clinical course was complicated by severe adverse events. During the first days of DA induction the patient suffered from acute dyspnea which led to his admission to the intensive care unit where he first received CPAP (Continuous Positive Airway Pressure) and then mechanical ventilation. A thoracic CT scan showed bilateral pleural effusions and atypical infiltrates. During these first days right-sided hemiplegia and desorientation developed. A cranial CT scan showed a left frontal and right pareto-occipital parenchymal bleeding. The renal function deteriorated and hemodialysis had to be performed. The clinical situation improved under broad-spectrum antibiotic treatment, the mechanical ventilation and hemodialysis could be discontinued and after 24 days on the ICU the patient was readmitted to the ward. The patient made a full recovery under intensive physical therapy with no residual neuro-psychiatric deficits. A bone marrow biopsy on day 15 after start of therapy showed a partial remission with around 10 to 20% blasts, and a second bone marrow biopsy on day 27, i.e. after leukocyte and platelet recovery, showed cytologically a suspicious residual blast population; however, flow cytometry did not confirm a significant blast population. The second induction cycle started on day 34 and had less adverse events. However, the patient developed febrile neutropenia and a methicillin-resistant *Staphylococcus aureus* (MRSA) was isolated from a blood culture. Despite receiving antibiotic treatment according to the resistogram, the patient developed pneumonic infiltrates and increasing C-reactive protein levels which only improved after a combination of voriconazole and liposomal amphotericin was added to the antibiotic regimen. After leukocyte recovery all symptoms rapidly resolved and the patient could be discharged with a prophylactic posaconazole medication. The therapy was continued with 3 consolidation cycles (MAC-MAMAC-MAC). Stem cells apheresis was performed after the second consolidation cycle. Complications during consolidation included reactivation of the pulmonary mycosis and a marked cervical lymphadenitis. Bone marrow cytology after each cycle showed a complete remission.

#### Assessment of minimal residual disease (MRD)

**Figure 2:**
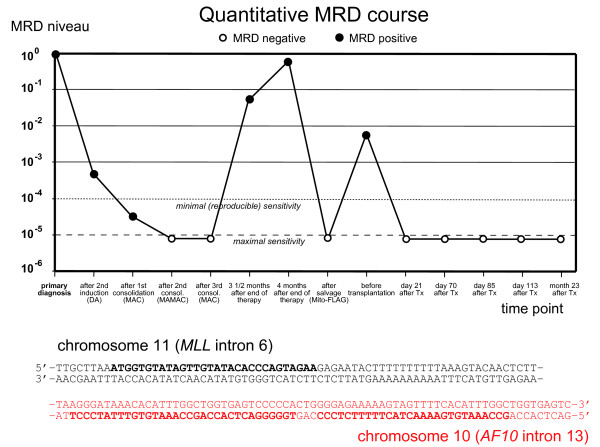
** Minimal residual disease (MRD) course of patient 1 over time.** Three further peripheral blood samples taken at months 25, 28 and 33 were also MRD-negative (not shown in this diagram). Below the chromosomal break region with PCR primers and hydrolysis probe indicated.

A patient-specific PCR for detecting minimal residual disease (MRD) was established as described previously [12]. The chromosomal breakpoint in the *MLL* gene was identified by long distance inverse PCR [13] and a highly sensitive quantitative PCR using a hydrolysis probe for detecting this breakpoint sequence was established [12] (Figure 2). The *maximal sensitivity* was 10^-5^ and the *reproducible sensitivity* was 10^-4^ in each case (terminology according to van der Velden [14]). This PCR showed continuously decreasing MRD which was below the detection limit after the second and third consolidation cycle (Figure 1).

#### Relapse and salvage therapy

Three and a half months after end of the therapy the patient presented with an inguinal swelling. A biopsy and a bone marrow investigation was undertaken. The inguinal biopsy revealed an extramedullary AML relapse. While bone marrow cytology appeared normal, MRD investigation showed MRD positivity (Figure 2). A second bone marrow investigation two weeks later confirmed the relapse. In addition, patchy skin infiltrates appeared. The patient was treated according to the Mito-FlAG scheme (2×15 mg/m^2^ fludarabine; 2×1000 mg/m^2^ cytarabine d1-5; 7 mg/m^2^ mitoxantrone d1,3,5). The treatment was relatively well-tolerated and besides a venous catheter-associated *S. epidermidis* bacteriaemia, no major complications occurred. After therapy the patient achieved complete cytomorphologic remission, the extramedullary manifestations had disappeared and no MRD was detectable in the bone marrow.

#### Haploidentical transplantation

**Figure 3:**
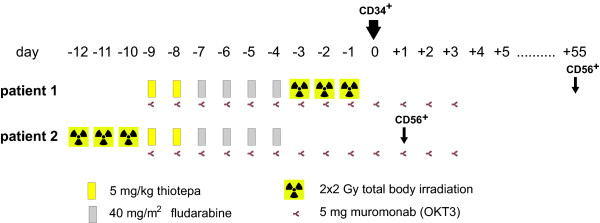
**Conditioning regimens for haploidentical transplantation.** For logistical reasons the irradiation was applied either before or after the chemotherapy. Stem cells were infused on day 0 in both patients. The second patients received donor-derived NK cells on day +1. In the first patient, NK cells were withheld until day +55 because of concerns regarding graft-versus-host disease due to the relatively high CD3+ cell content.

Since no HLA-identical donor was available, the haploidentical mother of the patient was chosen as stem cell donor. After successful eligibility assessment she underwent stimulation with filgrastim (2×10 μg/kg body weight) on days −5 to +1 and CD34^+^ stems cells were apheresed via an inguinal shaldon catheter on days 0 and +1. The patient was conditioned in parallel with 5 mg/kg thiotepa on day −9 and −8, 40 mg/m^2^ fludarabine on day −7 to −4 and fractionized 12 Gy total body irratiation on days −3 to −1 (Figure 3). Muromonab was given daily in a dose of 5 mg on days −9 to +3. The apheresed stem cells were T-cell depleted and 9.6 × 10^6^ CD34^+^ donor cells/kg recipient body weight were infused on days 0 and +1. The infusion of donor NK cells which had been scheduled for day +1 was postponed because of a relatively high CD3^+^ cell count (19.6 × 10^3^/kg = 1.5 × 10^6^ in total) and concern regarding severe graft-versus-host disease. The collected NK cells were archived in liquid nitrogen. The conditioning regime was tolerated relatively well. The clinical course after transplantation was characterized by infectious complications which could however be controlled with adequate antibiotic treatments, e.g. a CMV reactivation on day +27, which was successfully treated with valganciclovir. The leukocyte count was >1/nl since day +8 and the platelet count reached values >50/nl after day +13. No signs of GvHD grade >1 were noted after transplantation and thus it was decided to infuse the withheld donor NK cells on day +27. No GvHD occurred after this infusion.

#### Long-term follow-up and immune reconstituion

**Figure 4:**
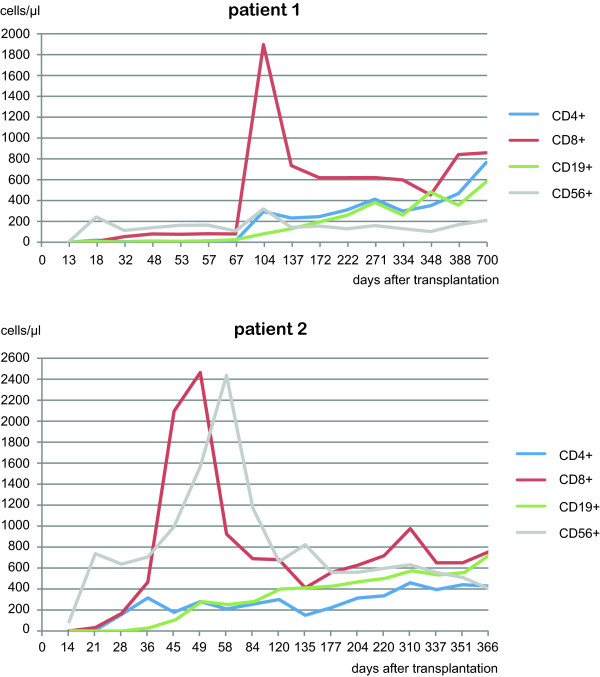
**Immune reconstitution after transplantation in terms of B cells **(**CD19**^**+**^)**, cytotoxic T cells (CD3**^**+**^**CD8**^**+**^**), T helper cells (CD3**^**+**^**CD4**^**+**^**) and NK cells (CD56**^**+**^**). **The first patient showed a sharp peak in CD8+ cells on day +104 (77 days after NK cell infusion) and the second patient showed the same phenomenon on day +49 (48 days after NK cell infusion), followed by a peak in NK cells. The first patient may also have had the NK peak, but it was possibly missed due to wider monitoring intervals. The peaks could not be correlated with clinical events.

The further clinical course was uneventful and required no hospitalizations. MRD follow-ups for almost three years showed no persistent leukemia and the patient returned to normal life working in his former profession and is now, 4 years after transplantation, clinically free of disease. Lymphocyte subpopulations were measured in the peripheral blood at different time points after transplantation (Figure 4). The T helper cell count was > 200/μl after day +104. Around day +104, a sharp increase in CD8^+^ cells was observed, however this could not be related to any clinical event. All cell subsets increased to normal values over time.

### Case 2

#### Features at presentation (relapse)

**Figure 5:**
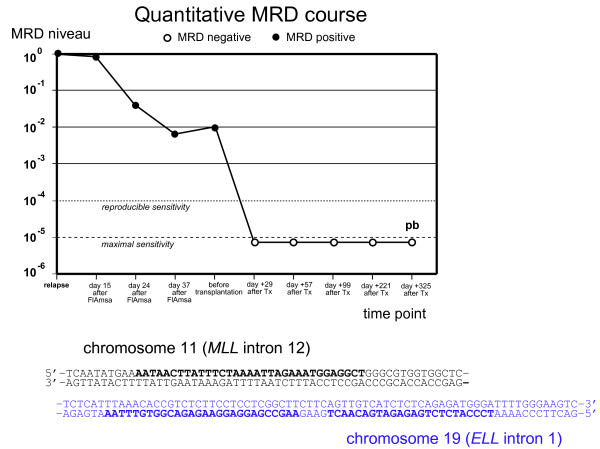
**Minimal residual disease (MRD) course of patient 2.** The last examined sample was taken from peripheral blood (pb). Below the chromosomal break region with primers and hydrolysis probe indicated.

A 28-year-old female patient was admitted to our hospital after diagnosis of relapsed acute myeloid leukemia. She had been diagnosed with AML FAB M2 16 months earlier and had been treated elsewhere according to the AML2003 protocol (see above) arm B with two cycles of DA induction therapy and 3 cycles of high-dose cytarabine (2 × 3 g/m^2^ d1,3,5) consolidation therapy. Cytological bone marrow examinations revealed a delayed blast clearance (persistence on day 15 of first induction, blast clearance after second induction). No HLA-identical sibling or unrelated donor was available. On presentation in our hospital she was in good overall condition. The blood count showed 3.3/nl leukocytes (30% blasts), 10.1 g/dl hemoglobin (0.29 hematocrit) and 79/nl platelets. The clinical chemical parameters and LDH were otherwise normal. Bone marrow examination confirmed the relapse (Figure 5). Cytogenetic analysis showed a t(11;19)(q23;q13.1) which had also been detected at the time of primary diagnosis. A *MLL-ENL* fusion transcript was detected by RT-PCR.

#### Salvage treatment and disseminated candidiasis

The patient was treated according to a modified FlAmsa protocol (30 mg/m^2^ fludarabine d1-4, 100 mg/m^2^ amsacrine d1-4, 2000 mg/m^2^ cytarabine). During the subsequent aplasia she developed an increase in C-reactive protein to around 30 mg/l (normal < 0.5 mg/l) despite broad-spectrum antibiotic treatment. A thoracic CT scan showed small bilateral pulmonary and hepatic lesions suggestive of atypical or fungal pneumonia. An MR scan of the abdomen showed hepatomegaly (22 cm craniocaudal diameter) with multiple lesions in liver, spleen and kidneys, measuring up to 8 mm in diameter. At the same time, an elevated Candida antigen level was detected in the serum. The patient was initially treated with liposomal amphotericin B and later with voriconazole. A bronchoalveolar lavage revealed 100–1.000 colony-forming units/ml *Candida dubliniensis* and a liver biopsy showed neither leukemic infiltrates nor fungi. *Candida dubliniensis* was later also isolated from a stool specimen. Systemic candidosis was diagnosed, the antimycotic treatment was changed to caspofungin and after a further increase in Candida antigen titer to micafungin with liposomal amphotericin B. The manifestation in lungs, liver, spleen and kidneys regressed and the C-reactive protein and the Candida antigen titer normalized after leukocytic regeneration. Liposomal amphotericin was discontinued 11 days before transplantation and micafungin was continued until day +31 after transplantation after which it was replaced by posaconazole. The candidiasis delayed the scheduled transplantation by about 4 weeks.

Minimal residual disease was assessed by quantifying the patient-specific chromosomal breakpoint sequence in the *MLL* gene as described in the first case. A real-time quantitative PCR was established and every bone marrow specimen was investigated for MRD. The MRD level before transplantation was 10^-2^ relative to the time of relapse (Figure 5).

#### Haploidentical transplantation

The younger brother of the patient was chosen as a suitable haploidentical stem cell donor. The conditioning regimen for haploidentical was similar to that described above but the 12 Gy total body irradiation was applied before the chemotherapy for logistical reasons (Figure 1). A bone marrow investigation shortly before start of conditioning revealed an MRD level of 10^-2^, which was only slightly higher compared to that achieved one month ago after FlAmsa therapy (Figure 1). The patient received a high stem cell dose (15.87 × 10^6^/kg body weight) from the donor (Tables 1, 2, and 3). On day +1 after CD34^+^ cell transplantation donor NK cells (5.46 × 10^6^/kg) were infused. The clinical course after transplantation was relatively uncomplicated. In particular, there were no signs of recurrence of invasive fungal infection under continuous daily micafungin medication. The patient showed a mild graft-versus-host reaction (grade 1) of the skin and intestine (both not histologically verified) which regressed without immunosuppression. Clinical engraftment of leukocytes (>1/nl) and platelets (>50/nl) occurred on day +10 and +13.

**Table 1:** Immunogenetic characteristics (HLA and KIR genotypes)

	**Recipient HLA**	**Donor HLA**	**Recipient KIR**	**Donor KIR**
**case 1**	A*0101, A*0301, B*1801, B*4101, Cw*0102, Cw*1701, DRB1*0301, DRB1*1301, DQB1*0201, DQB1*0603	A*0101, A*2902, B*1801, B*4403, Cw*0102, Cw*1601, DRB1*0301, DRB1*0701, DQB1*0201, DQB1*0202	2DL1+, 2DL2+, 2DL3-, 2DL4+, 2DL5+, 2DP1+, 2DS1 +, 2DS2+, 2DS3+, 2DS4-, 2DS5-, 3DL1+, 3DL2+, 3DL3+, 3DP1+, 3DS1+ (2DS4 could not be excluded).	2DL1+, 2DL2+, 2DL3+, 2DL4+, 2DL5-, 2DP1+, 2DS1-, 2DS2+, 2DS3-, 2DS4+, 2DS5-, 3DL1+, 3DL2+, 3DL3+, 3DP1+, 3DS1+
**case 2**	A*0201, A*2601, B*4403, B*5701, Cw*0401, Cw*0602, DRB1*0402, DRB1*0701, DQB1*0201, DQB1*0302	A*0201, A*6801, B*1302, B*4402, Cw*0501, Cw*0602, DRB1*0401, DRB1*0701, DQB1*0201, DQB1*0301	2DL1+, 2DL2-, 2DL3+, 2DL4+, 2DL5+, 2DP1+, 2DS1+, 2DS2-, 2DS3-, 2DS4+, 2DS5+, 3DL1+, 3DL2+, 3DL3+, 3DP1+, 3DS1+	2DL1+, 2DL2-, 2DL3+, 2DL4+, 2DL5+, 2DP1+, 2DS1+, 2DS2-, 2DS3-, 2DS4+, 2DS5+, 3DL1+, 3DL2+, 3DL3+, 3DP1+, 3DS1+

Patient 2 was serologically HLA-haploidentical to her donor. However, high resolution DNA typing and HLA typing of the patient’s father revealed that donor and patient had inherited different HLA haplotypes from their parents and were coincidentially nearly haploidentical (haploidentity apart from one molecular B44 mismatch).

**Table 2:** **CD34**^**+**^**cells transplant characteristics**

	**Total WBC (10**^**6**^**)**	**CD34**^**+**^**count (10**^**6**^**), total and per kg**	**CD34**^**+**^**purity**	**CD3**^**+**^**count (10**^**3**^**), total and per kg**	**CFU (10**^**5**^**/kg)**
**case 1**	770	753 (9.6/kg)	97.8%	548 (7.0/kg)	13.7
**case 2**	833	825.5 (15.87/kg)	99.5%	374 (8.0/kg)	28.2

**Table 3:** NK cell doses

	**Total WBC (10**^**6**^**)**	**CD56**^**+**^**count (10**^**6**^**), total and per kg**	**CD56**^**+**^**content**	**CD3**^**+**^**count (10**^**3**^**), total and per kg**
**case 1**	659	337 (4.32/kg)	51%	1530 (19.6/kg)
**case 2**	375	284.5 (5.46/kg)	91.6%	740 (14.0/kg)

#### Immune reconstitution and MRD after transplantation

The MRD level was continuously negative in all investigations after transplantation during the follow-up period of more than one year (Figure 5). Immune reconstitution occurred relatively fast, and T helper cells first reached a level of >200/nl on day +36. A peak in CD8^+^ cells was observed on day +49 followed by a peak in CD8^+^ cells on day +58.

## Conclusions

### Haploidentical transplantation and the possible role of KIR mismatches

The difficulties in finding a suitable HLA-identical donor for every patient in need for it led to the idea of considering the “always present but overlooked” [6] haploidentical relatives as potential donors. The patient’s parents and children are always HLA-haploidentical and the probability of a patient’s sibling being HLA-haploidentical is 50% in contrast to 25% probability for full HLA identity. Thus in practical reality the probability of finding a HLA-haploidentical relative is nearly 100%.

Two major problems have to be overcome in haploidentical transplantation: the first is graft rejection and the second is severe graft-versus-host disease. The group of Martelli et al*.* first demonstrated clinically that this is possible by using large amounts (“megadoses”, i.e. > 10^7^/kg) of highly purified CD34^+^ hematopoietic stem cells. These purified stem cells are largely devoid of T-cells which are responsible for the graft-versus-host effect.

To achieve some kind of alloreactivity against residual recipient’s blood cells, donor-derived natural killer (NK) cells have been infused. The basis of this NK alloreactivity is not fully understood. NK cells are able to attack other cells that show downregulation of HLA class I molecules on their surface which is often the case in virus-infected cells or tumor cells. However, HLA class I downregulation is neither necessary nor sufficient for NK cell activation as is illustrated by the fact that normal cells or tissues lacking HLA class I expression (e.g., erythrocytes) or showing low HLA class I expression (e.g., neural cells) are not attacked by autologous NK cells. Cytotoxic NK cell activity is believed to be mainly determined by the presence of inhibitory or activating receptors on the NK cell surface. Two major types of inhibitory receptors have been identified: killer cell immunoglobulin-like (KIR) receptors and the CD94/NKG2A heterodimer, a C-type lectin, which interacts with the non-classical class Ib HLA-E molecule (reviewed in [15]). While the ligands for many KIR receptors are unknown, the inhibitory receptors KIR2DL1, KIR2DL2 and KIR3DL1 bind epitopes of HLA-C (’HLA-C2, -C1’ groups) and HLA-A and HLA-B molecules with ’Bw4” motif, respectively [16]. Upon encountering self-class HLA antigen, NK cell–mediated lysis and cytokine release are inhibited.

While HLA genes are located in the MHC complex on chromosome 6p21.3, KIR genes are clustered in the leukocyte receptor complex (LRC) on chromosome 19q13.4. Thus they segregate independently in human pedigrees. The LRC is highly polymorphic and contains seven to 15 KIR gene loci dispersed within a region of 65 to 200 kb [17]. It is not yet fully understood how NK cells are “educated” not to attack other autologous cells lacking inhibitory HLA ligands for their KIR receptors [18,19]. Various models have been proposed to explain the experimentally observed alloreactivity of NK cells. The “licensing model” predicts that NK cells that do not receive a signal through an inhibitory receptor become hyporesponsive instead of alloreactive. The “Ruggieri model” assumes that NK cells with inhibitory KIR receptors for ligands absent on both donor and recipient are nonalloreactive. The effect of NK cell alloreactivity in the setting of a fully HLA-matched transplantation has been controversially discussed (synopsis in [16]). Some groups have found a benefit in overall survival in case of a KIR-HLA class I mismatch [20], but others could not confirm this and found no effect [21] or even a worse outcome [22]. In haploidentical transplantation the Perugia group has published evidence that NK cells with a mismatch between donor KIR and recipient's HLA molecules in graft-versus-host direction positively correlate with outcome at least in patients with myeloid leukemias [10,11].

Both our patients and their donors showed inhibitory and activating KIR genes corresponding to KIR haplotype group B. KIR ligands and mismatches were calculated using the EMBL IPD-KIR database release 2.4.0 [23]. Both donors were KIR3DL1-positive but only the second patient had the corresponding inhibitory receptor Bw4 (B44 and B57 with Bw4). The donor of patient 1 expressed KIR2DL2, 2DL3, 2DS2, 2DL1 and 2DS2, all of which are ligands for Cw1 und Cw17, which were expressed by patient 1. The first patient showed mismatches in both directions, a mismatch in GvH direction (donor KIR3DL1-based) and a mismatch in HvG direction (recipient KIR2DL1-based). The second patient showed neither a mismatch in GvH nor HvG direction. Since no functional tests were performed it was not possible to assess any NK cell-based alloreactivity in both cases.

### Minimal residual disease before and after transplantation

Due to the extraordinary genetic heterogeneity of AML, it is difficult to set up general recommendations for molecular monitoring of residual disease (recently reviewed by [24-26]). Astonishingly few studies have addressed the issue of MRD monitoring before and after allogeneic transplantation in AML. Although the same MRD levels may have a different prognostic impact in “good risk” and “bad risk” AML, there is little doubt that the level of molecular remission is in principle of critical importance for the probability of long-term remission and the risk of relapse. The issue how often and when to assess MRD in AML is still controversial, but attempts have been made to provide general recommendations for MRD monitoring [24]. Basically two different methods have been used for MRD assessment, polymerase chain reaction (PCR) and flow cytometry. The main advantage of flow cytometry is the speed of getting results; however, disadvantages of the method are the lack and principal difficulties in standardization. The theoretical maximum achievable sensitivity is higher for flow cytometry than PCR, but in reality it is often significantly lower. To obtain a higher sensitivity, multiparameter flow has to be used, but this again makes the procedure more complicated and investigator-dependent. PCR is based on the quantitative detection of clonal markers which are often, but not necessarily, also disease markers, such as leukemogenic fusion genes. In lymphoid neoplasms the clonally rearranged immune genes of the malignant clone can often be used as an MRD target. They are no disease but surrogate markers and may sometimes get lost during the course of disease due to further immune gene rearrangements. Detection of MRD by PCR can be based on RNA or DNA targets. RNA targets, such as chimeric transcripts originating from leukemogenic fusion genes, can often be assessed more easily, but MRD detection based on such RNA molecules has the disadvantage that no absolute quantification is possible. Instead the quantification is calculated relative to a “housekeeping gene” or “control gene” which is supposed to be stably expressed; of course this can only be an approximation. Besides that, RNA is relatively unstable and degrades easily if not stored at low temperatures. In contrast, DNA is remarkably stable, usually over several days at room temperature. PCR methods based on the quantification of DNA targets allow a precise absolute quantification of the target. It is not trivial to find such a clonal PCR target in every case of AML. However, in acute leukemia with *MLL* translocation it is possible and feasible to use the chromosomal breakpoint in the *MLL* gene as a molecular marker for PCR [12]. The resulting PCR usually has a very high sensitivity and stability. The *MLL* breakpoint can relatively easily and elegantly be identified using a generic inverse PCR method [13].

Sixteen AML patients at our institution received a haploidentical stem cell transplant in the time period between 2004 and 2011, mostly because no suitable HLA-identical donor was available, but in a few cases also because of relapse after HLA-identical stem cell transplantation. Fifteen out of the sixteen patients were transplanted with active disease/relapse at transplantation. The overall survival was 24% with a median follow-up of 3.7 years (detailed data soon to be published). Several factors may have contributed to the favourable outcome despite several risk factors in our two patients. First, both were relatively young and had virtually no comorbidities. Second, in spite of early relapses, the leukemias were still chemotherapy-sensitive and responded relatively well to salvage therapy. Third, the MRD niveau before transplantation was not very high. A high pre-HCT MRD level has been associated with an increased risk of relapse after myeloablative HCT for AML [27]. Last but not least the regular MRD assessments were certainly helpful to guide therapeutical decisions during the course of disease.

In short summary, the two cases presented here illustrate the value of regular MRD monitoring in the pre- and post-transplant setting for guiding clinical decisions. They show exemplarily the immune reconstitution after haploidentical transplantation and illustrate that under certain circumstances haploidentical transplantation may be an option even for patients with a history of invasive fungal infections.

### Consent

Written informed consent was obtained from the patients for publication of this case report and any accompanying images. A copy of the written consent is available for review by the Editor-in-Chief of this journal.

## Competing interests

The authors declare that they have no competing interests.

## Authors' contributions

TB: wrote the manuscript, designed MRD method, treated patients. MM: performed technical PCR work. CM and RM: obtained and analyzed genomic chromosomal breakpoint sequences. NL: performed HLA and KIR typing. SS: performed bone marrow and flow cytometry analysis. BF, IWB, JB, LU (head of stem cell facility): treated patients, analyzed data. GL: planned and supervised radiation therapy. CTB: obtained and processed stem cells and NK cells. All authors read and approved the final manuscript.
